# Screening for new antiretroviral targets

**DOI:** 10.1186/1742-4690-10-S1-O42

**Published:** 2013-09-19

**Authors:** Ariberto Fassati

**Affiliations:** 1 Wohl Virion Centre, Department of Infection, MRC Centre for Medical Molecular Virology, University College London, 90 Gower Street, London WC1E 6BT, UK

## Background

Viruses are obligate intracellular parasites, which must exploit cellular pathways and specific factors to replicate. Many fundamental discoveries were made studying interactions between host and virus, including the discovery of interferon, intrinsic immunity, oncogenes, mRNA splicing, RNA interference, and tRNA retrograde transport in human cells. Detailed knowledge of host-pathogen interactions also fosters the development of new therapeutic strategies. Specific targeting of host factors has led to new drugs to inhibit HIV-1 infection being introduced in the clinic or about to enter clinical trials.

## Methods

We performed chemical genetic screenings using libraries of small molecules and HIV-1 vectors. Hits were identified by at least two-fold inhibition of infection in the absence of toxicity. Validation included dose-response curves, assessment in primary cells with replication competent HIV-1, target knock down, and isolation of escape mutant viruses.

## Results

The gyrase B inhibitor Coumermycin A1 (CA1) was a confirmed hit, which inhibited HIV-1 replication by independently targeting the viral capsid protein and Hsp90. CA1 targeted a specific pocket in capsid, as determined by mapping a viral escape mutant, isothermal titration calorimetry and molecular docking. CA1 induced faster virus uncoating, which resulted in lower accumulation of capsid in the nucleus of infected cells and reduced integration efficiency. Targeting of Hsp90 with CA1 impaired HIV-1 gene expression in acutely infected cells. Using chromatin immunoprecipitation (ChIP) and immunofluorescence, Hsp90 was found to migrate to the viral promoter. Hsp90 also mediated enhanced viral replication in conditions of hyperthermia. Inhibition of Hsp90 specifically impaired the NFkB pathway and prevented HIV-1 reactivation from latency.

## Conclusions

From a single hit in our screening, we have found that HIV-1 capsid is a novel target influencing integration efficiency, and that Hsp90 is a key mediator required for HIV-1 gene expression and reactivation from latency.

